# Two new species and a new subgenus of toothed *Brachyhypopomus* electric knifefishes (Gymnotiformes, Hypopomidae) from the central Amazon and considerations pertaining to the evolution of a monophasic electric organ discharge

**DOI:** 10.3897/zookeys.327.5427

**Published:** 2013-08-28

**Authors:** John P. Sullivan, Jansen Zuanon, Cristina Cox Fernandes

**Affiliations:** 1 Cornell University Museum of Vertebrates, 159 Sapsucker Woods Road, Ithaca, New York 14850 USA; 2 Instituto Nacional de Pesquisas da Amazônia, Coordenação de Biodiversidade, Avenida André Araújo 2936, CEP 69080-971, Manaus, Brazil; 3 Biology Department, Morrill Science Center, University of Massachusetts, Amherst, Massachusetts 01003 USA

**Keywords:** Gymnotiform, weakly electric fish, electric organ, electric organ discharge, EOD, Neotropical freshwater fishes, evolution

## Abstract

We describe two new, closely related species of toothed *Brachyhypopomus* (Hypopomidae: Gymnotiformes: Teleostei) from the central Amazon basin and create a new subgenus for them. *Odontohypopomus*, new subgenus of *Brachyhypopomus*, is diagnosed by (1) small teeth present on premaxillae; (2) medialmost two branchiostegal rays thin with blades oriented more vertically than remaining three rays; (3) background color in life (and to lesser extent in preservation) distinctly yellowish with head and sides peppered with small, widely spaced, very dark brown stellate chromatophores that greatly contrast with light background coloration; (4) a dark blotch or bar of subcutaneous pigment below the eye; (5) electric organ discharge waveform of very long duration (head-positive phase approx. 2 milliseconds or longer, head-negative phase shorter or absent) and slow pulse repetition rate (3–16 Hz). The type species of the new subgenus, *Brachyhypopomus (Odontohypopomus) walteri* sp. n., is diagnosed by the following additional character states: (1) subcutaneous dark pigment at base of orbit particularly prominent, (2) body semi-translucent and nearly bright yellow background coloration in life, (3) a biphasic electric organ discharge (EOD) waveform of very long duration (between 3.5 and 4 milliseconds at 25° C) with head-positive first phase significantly longer than second head-negative phase in both sexes. *Brachyhypopomus (Odontohypopomus) bennetti* sp. n. is diagnosed by two character states in addition to those used to diagnose the subgenus *Odontohypopomus*: (1) a deep electric organ, visible as large semi-transparent area, occupying approximately 14–17% body depth directly posterior to the abdominal cavity in combination with a short, but deep, caudal filament, and (2) a monophasic, head-positive EOD waveform, approximately 2.1 milliseconds in duration in both sexes. These are the only described rhamphichthyoid gymnotiforms with oral teeth, and *Brachyhypopomus bennetti* is the first *Brachyhypopomus* reported to have a monophasic (head-positive) EOD waveform. Unlike biphasic species, the waveform of its EOD is largely unaffected by tail damage from predators. Such injuries are common among specimens in our collections. This species’ preference for floating meadow habitat along the major channels of the Amazon River basin may put it at particularly high risk of predation and “tail grazing.”

## Abstract (pt)

Nós descrevemos duas novas espécies proximamente relacionadas de *Brachyhypopomus* (Hypopomidae: Gymnotiformes: Teleostei) da porção central da bacia Amazônica e criamos um novo subgênero para elas. *Odontohypopomus*, novo subgênero de *Brachyhypopomus*, é diagnosticado por (1) apresentar dentes pequenos no pré-maxilar; (2) pela forma dos dois raios branquiostegais mais internos, com expansão laminar fina orientada mais verticalmente do que nos três raios remanescentes; (3) coloração de fundo em vida (e, em menor grau, após preservação) distintamente amarelada, com a cabeça e flancos marcados por pequenos cromatóforos marrom escuro de formato estelar e bem espaçados entre si, contrastando fortemente com a tonalidade clara de fundo; (4) uma mancha ou barra escura de pigmento subcutâneo abaixo do olho; (5) forma da onda de descarga do órgão elétrico (DOE) de duração muito longa (fase positiva com aproximadamente 2 milisegundos ou mais, fase negativa mais curta ou ausente) e uma baixa taxa de repetição de pulsos (3–16 Hz). A espécie-tipo do novo subgênero, *Brachyhypopomus (Odontohypopomus) walteri* sp. nov., é adicionalmente diagnosticada pelos seguintes estados de caráter: (1) pigmentação subcutânea na base da órbita particularmente conspícua; (2) corpo semi-translúcido e com coloração geral em vida amarelo forte, (3) DOE com onda bifásica e de duração muito longa (entre 3,5 e 4 milisegundos a 25° C), com a fase inicial positiva significativamente mais longa do que a segunda fase (negativa) em ambos os sexos. *Brachyhypopomus (Odontohypopopmus) bennetti* sp. nov. é diagnosticada por dois estados de caráter, além daqueles utilizados para diagnosticar o subgênero *Odontohypopomus*: (1) órgão elétrico largo em vista lateral, visível como uma grande área semitransparente ocupando aproximadamente 14–17% da altura do corpo, medida imediatamente posterior à cavidade abdominal, combinada com um filamento caudal curto e alto; e (2) DOE monofásica positiva, com duração de aproximadamente 2,1 milisegundos em ambos os sexos. Esses são os únicos Gymnotiformes Rhamphichthyoidea com dentes orais e *Brachyhypopomus bennetti* é a primeira espécie de *Brachyhypopomus* conhecida por apresentar uma DOE monofásica positiva. Diferentemente das espécies com DOE bifásica, a forma de onda/pulso dessa espécie não é significantemente afetada por danos ao filamento caudal decorrentes de predação. Esse tipo de dano é comum entre os espécimes examinados em nossas coleções. A ocorrência predominante dessa espécie em bancos flutuantes de macrófitas ao longo de alguns dos principais rios formadores da Bacia Amazônica pode representar um risco particularmente alto de predação e mutilação do filamento caudal.

## Introduction

Hypopomid knifefishes are nocturnally active, invertivorous inhabitants of lentic and slowly flowing freshwater habitats from Panama to Uruguay. While infrequently exploited for human consumption because of their small size, hypopomids are often abundant and ecologically important components of Neotropical freshwater environments (Hagedorn 1988, Westby 1988, Crampton 1996, 1998, 2011). The family Hypopomidae is one of six nominal families within the Order Gymnotiformes, all of which detect nearby objects and communicate by means of an active electrosensory system in which weak autogenic electric fields are monitored by frequency-matched dermal electroreceptors. Only the gymnotiform electric eel, *Electrophorus electricus*, has the additional capacity to produce strong electric potentials for prey capture and defense (Zupanc and Bullock 2005). The pulsatile electric organ discharges (EODs) of hypopomids often have species-specific characteristics that are useful for their taxonomy and even identification in the field. Such is particularly the case for the two new species of hypopomid gymnotiform we describe here that frequently co-occur in “floating meadow” habitat common along marginal lagoons and channels of the central Amazon basin.

*Brachyhypopomus* was created by Mago–Leccia (1994) to distinguish *Hypopomus brevirostris* (Steindachner, 1868) and other species with short snouts from the longer-snouted *Hypopomus artedi* (Kaup, 1856) and is one of seven genera recognized within the family Hypopomidae (Albert and Crampton 2003). Mago–Leccia (1994) designated *Brachyhypopomus brevirostris* (Steindachner, 1868), described from the Río Guaporé (Amazon basin) of Bolivia, as the type species of the genus and recognized an additional five species: *Brachyhypopomus occidentalis* (Regan, 1914) from the Río Condoto (Pacific slope) of Colombia, *Brachyhypopomus beebei* (Schultz, 1944) from the Río San Juan (Caribbean drainage) of Venezuela, *Brachyhypopomus diazi* (Fernández Yépez, 1972) from the Río Alpargatón (Caribbean drainage) of Venezuela, *Brachyhypopomus pinnicaudatus* (Hopkins, 1991) from the coastal swamps of French Guiana, and *Brachyhypopomus janeiroensis* (Costa & Campos da Paz, 1992) from the Rio São João near Rio de Janeiro, Brazil. Since then, five additional *Brachyhypopomus* species have been described. *Brachyhypopomus bullocki* Sullivan & Hopkins (2009) is from the Llanos (Orinoco basin) of Colombia and Venezuela; the other four species are from the southernmost part of the range of gymnotiforms in southeastern Brazil and Uruguay: *Brachyhypopomus jureiae* Triques & Khamis (2003), *Brachyhypopomus bombilla* Loureiro & Silva (2006), *Brachyhypopomus draco* Gioria et al. (2008), and *Brachyhypopomus gauderio* Gioria & Malabarba (2009). The two *Brachyhypopomus* species treated here are the first to be described from the central Amazon, although *Brachyhypopomus brevirostris*, *Brachyhypopomus beebei*, *Brachyhypopomus pinnicaudatus*, *Microsternarchus bilineatus* and other undescribed hypopomids co-occur with them in this region (Sullivan 1997, Albert and Crampton 2003).

Morphological characters that unequivocally support the monophyly of *Brachyhypopomus* are few. Sullivan (1997) listed only a single possible synapomorphy for *Brachyhypopopmus*: the anterior portion of the maxilla is curved such that sides of the upper jaw descend at a distinct angle from the medial (premaxillary) portion of the upper jaw. In most rhamphichthyoids, the maxilla is straight to slightly curved, and the medial and lateral portions of the upper jaw form a continuous curve with little to no inflection point, viewed externally. However, this form of maxilla also occurs in the short-snouted rhamphichthyoid genus *Steatogenys* and in the Family Sternopygidae and may just be a concomitant feature of short (as opposed to more tubular) snouts. Albert (2001) recognized a monophyletic group consisting of the *Brachyhypopmus* species recognized by Mago–Leccia (1994) and several undescribed forms on the basis of four synapomorphies: (1) premaxilla gracile with a curved anterior margin and forming a distinct angle with the maxilla in lateral view, (2) dentary gracile, (3) body cavity with 16 or 17 precaudal vertebrae, and (4) a single transitional vertebrae. We regard these characters in combination with those enumerated by Mago-Leccia (1994) as provisionally sufficient to diagnose *Brachyhypopomus*, with the exception that pre-caudal vertebrae may be fewer than indicated by Albert: *Brachyhypopomus bullocki* Sullivan & Hopkins (2009) has a short abdominal cavity with only 11–13 precaudal vertebrae. An unpublished phylogenetic analysis of mitochondrial DNA sequences in Sullivan (1997) indicated monophyly of eleven species, seven of which are now treated as valid *Brachyhypopomus*, with four additional undescribed forms, two of which are those treated here. This group of eleven species is monophyletic with respect to *Hypopomus artedi*, *Microsternarchus bilineatus*, and species of *Hypopygus*, *Steatogenys*, *Rhamphichthys* and *Gymnorhamphichthys* (Sullivan 1997).

Within gymnotiforms the complete absence of oral teeth is a character state unique to the Hypopomidae and Rhamphichthyidae and is among those used to unite these two families into the superfamily Rhamphichthyoidea (Mago–Leccia 1976, 1978, 1994, Triques 1993, Sullivan 1997, Albert 2001, Albert and Crampton 2003). The two species described here are remarkable for being the only rhamphichthyoids known to bear premaxillary teeth.

## Materials and methods

Fishes were collected during day trips from Manaus, Brazil in a motorboat between March and May 1993; others were collected during the Calhamazon Project (Cox Fernandes et al. 2004) in November and December of the same year. The primary collection site for the type material is a few kilometers due south of Manaus in a series of channels, shallow lakes and islands that lie between the blackwater Rio Negro and the whitewater Rio Solimões, close to their confluence, as well as the Ilha da Marchantaria, a large, seasonally flooded island in the Solimões itself. We transported freshly captured individuals to a laboratory at the Instituto Nacional de Pesquisas da Amazônia(INPA) in Manaus with water from their capture locality. We recorded their EODs in a 10 cm x 40 cm x 12 cm aquarium with silver/silver–chloride electrodes positioned at the head (positive electrode) and tail (negative electrode) of the fish and a reference electrode in the center. EODs were amplified using a CWE Corporation bio–amplifier with filters set to 0.1 Hz to 50,000 Hz using low gain and captured with a Tektronix 222 digital storage oscilloscope (512 point/8-bit resolution). Longer recordings of EOD trains from the specimens here designated as holotypes were recorded on a Sony Walkman Pro cassette tape recorder, later digitized at 48 kHz on an Edirol FA-66 (Roland Corporation, Los Angeles, CA) and written to wav files with Audacity 2.0 software. Specimens were overanesthetized in MS-222, photographed in a photo aquarium, tagged, and fixed in 10% buffered formalin. Procedures for handling, euthanizing and preserving fishes followed guidelines for the use of fishes in research (AFS/ASIH/AIFRB 1987).

We examined thetype material for all described *Brachyhypopomus* species with the exception of those recently described from southern Brazil and Uruguay (*Brachyhypopomus jureiae*, *Brachyhypopomus bombilla*, *Brachyhypopomus draco*, and *Brachyhypopomus guaderio*) for which we consulted the published descriptions. We also examined a large quantity of non–type material of both described and undescribed forms (see Comparative Material Examined, below). Measurements were taken with a digital, needle–point caliper to within 0.1 mm under low power magnification. All measurements were taken point–to–point, i.e. not orthogonal to the main body axis. Counts of anal–fin rays and vertebrae were made from film radiographs of the specimens, observed under magnification. All vertebral counts began with C5, the first vertebra to bear a neural spine. “Precaudal vertebrae” include all anterior vertebrae bearing neural spines up to the first vertebra to bear a hemal spine. Vertebrae bearing hemal spines are termed “caudal vertebrae.” Counts of pectoral-fin rays, made with the aid of dissecting microscope and strong transmitted light, include all elements. Measurements were taken on the left side unless otherwise specified.

Anatomical measurements and abbreviations follow Hubbs and Lagler (1958). Three measurements require explanation. (1) LEA is the length from the tip of the snout to the posterior end of the anal-fin base. This measurement is generally used as standard length in descriptions of gymnotiforms, since most lack caudal fins and often have regenerated a portion of their caudal filament after injury from predators. Specimens that had suffered such damage anterior to the terminus of the anal fin were identified from radiographs, and excluded from measurements involving the length of the body reported for the type series. LEAs reported on damaged individuals are noted as such. (2) Head length(HL) is measured from the tip of the snout to the end of the opercle bone, not to the uppermost limit of the branchial membrane. (3) Interorbital width is the distance between the upper margins of the eyes. The term “branched” pectoral-fin rays refers to all rays posterior to the anterior unbranched rays, even if the posterior terminal ray is unbranched. The abbreviation “alc” is used to indicate specimens that are preserved in alcohol, “cs” for those that have been cleared and stained. Institutional abbreviations follow Sabaj Pérez (2012).

Because measuring body depth is problematic due to lack of external landmarks on these fishes, distance from the tip of the snout to the 1^st^, 20^th^ and 40^th^ caudal vertebrae were obtained from film radiographs of these specimens. These distances were then measured off on the specimens themselves and the depth of the body at each of these three points was obtained with digital needle point calipers. Measurements are presented as percentages of LEA except for those within the head that are presented as percentages of HL.

Staining protocols for bone and cartilage followed Potthoff (1984). In order to count columns of electrocytes in preserved specimens, skin was peeled back from caudal filaments and electrocytes were observed with strong transmitted light. “Electrocyte columns” refers to the number of bilateral bands of electrocytes along the longitudinal axis of the fish that begin under the head and continue to the tip of the caudal filament. These bands are most visible just above the posterior anal–fin base and on the caudal filament. These columns can often be counted without special preparation by viewing the area with strong transmitted light.

## Systematics

### Family Hypopomidae Mago-Leccia, 1978
Genus *Brachyhypopomus* Mago-Leccia, 1994

#### Subgenus Odontohypopomus subgen. n.

http://zoobank.org/7AFCDABC-A141-4E89-B72E-964D17137C5D

http://species-id.net/wiki/Odontohypopomus

##### Type species.

*Brachyhypopomus (Odontohypopomus) walteri* sp. n.

##### Included species.

*Brachyhypopomus (Odontohypopomus) walteri* sp. n., *Brachyhypopomus (Odontohypopomus) bennetti* sp. n.

**Figure 1.**
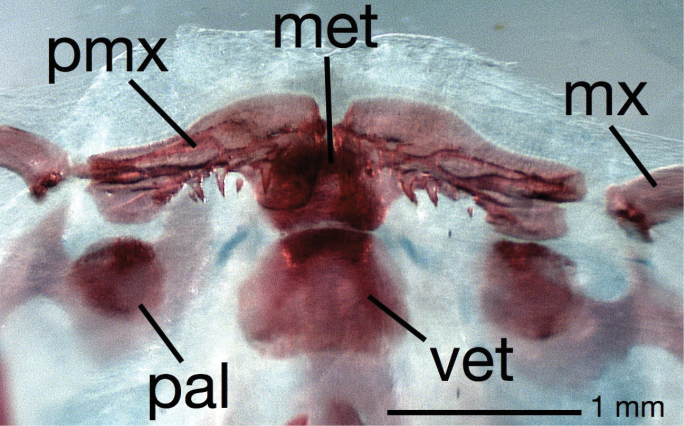
Upper jaw in ventral view of cleared and stained specimen of *Brachyhypopomus (Odontohypopomus) walteri* sp. n. (ANSP 194025) showing teeth present on the premaxillae. **PMX**=premaxilla, **MX**=maxilla, **MET**=mesethmoid, **PAL**=ossified element within palatine cartilage (not stained), **VET**=ventral ethmoid.

**Figure 2.**
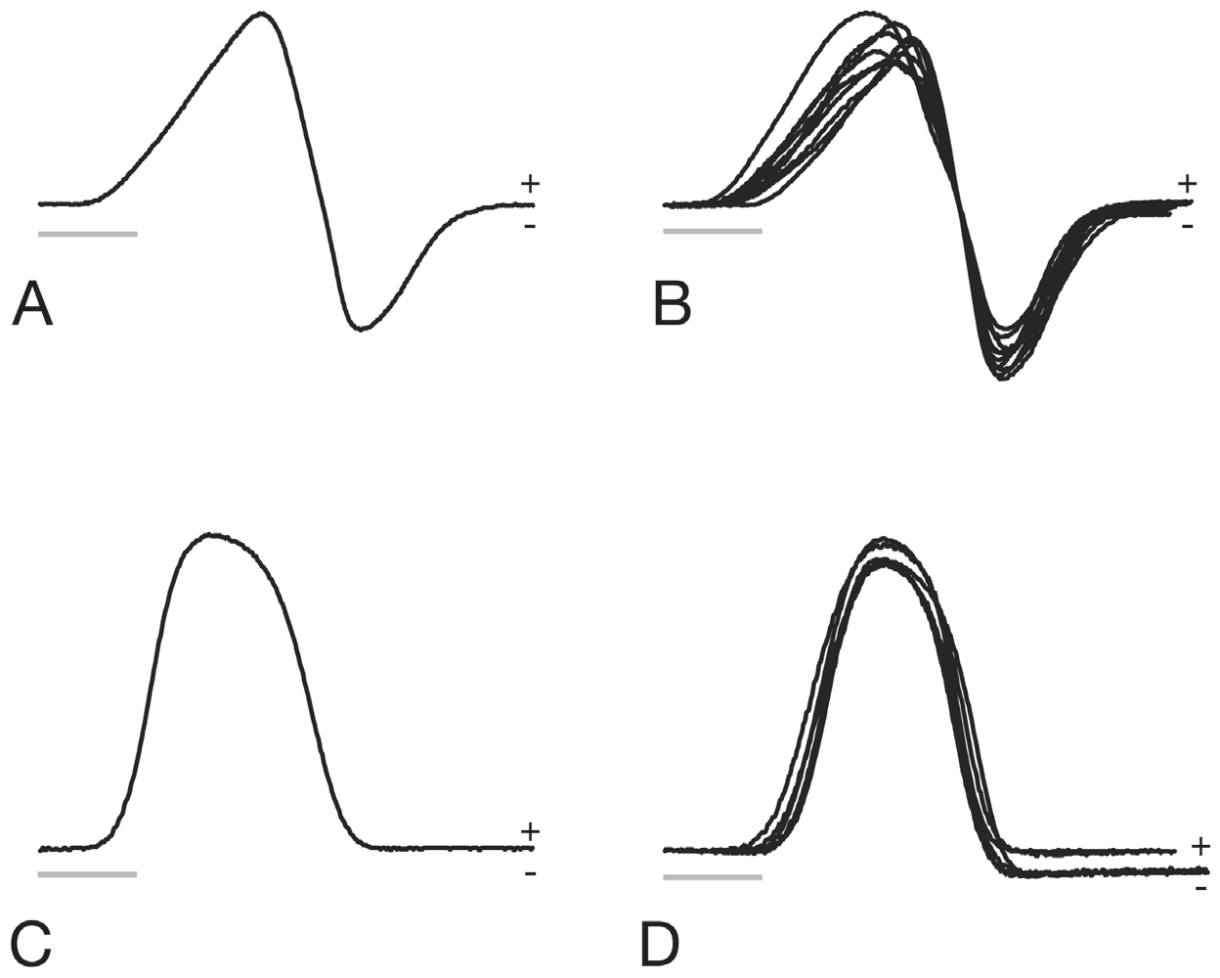
Electric organ discharge (EOD) waveforms of *Brachyhypopomus walteri* sp. n.and *Brachyhypopomus bennetti* sp. n. **A** EOD of holotype specimen of *Brachyhypopomus walteri* sp. n. **B** EODs of nine paratypes of *Brachyhypopomus walteri* sp. n. **C** EOD of holotype of *Brachyhypopomus bennetti* sp. n. **D** EODs of six paratypes of *Brachyhypopomus bennetti* sp. n. All are five millisecond traces with head positivity recorded upwards; water temperature between 21 and 23°C. Scale bars = 1 millisecond.

**Diagnosis.** This subgenus of *Brachyhypopomus* is diagnosed by (1) teeth present on premaxillae: usually one to five small, needle-like teeth on ventral surface of each (Fig. 1); (2) medialmost two branchiostegal rays thin with blades oriented more vertically than remaining three rays; (3) background color in life and to lesser extent in alcohol distinctly yellowish, head and sides peppered with small, widely spaced, dark brown stellate chromatophores that greatly contrast with background color of skin; bands along sides poorly defined, saddles of pigment mostly incomplete over dorsum; (4) a diffuse blotch of subcutaneous pigment directly beneath orbit, suggestive of a teardrop; (5) EOD pulse waveform of very long duration (head-positive phase approx. 2 milliseconds or longer, head-negative phase shorter or absent; Fig. 2) and slow repetition rate (3–16 Hz).

Teeth are absent from the premaxillae in all other rhamphichthyoid species, but present in all other gymnotiform lineages. (The teeth in preserved *Odontohypopomus* tend to be obscured by overlying tissue and are only easily visible in cleared and stained specimens.) In other *Brachyhypopomus*, the first (more medial) one or two branchiostegal rays are wide and oriented nearly horizontally, EODs are of shorter duration and faster repetition rates with second head–negative phases nearly equal in amplitude or of greater duration than the head–positive first phase, teardrop-like pigment below the orbit is absent, background color is less yellowish and chromatophores not as dark. In several other *Brachyhypopomus* species the pigment on the anterior flanks is arranged into distinct bands.

##### Etymology.

A combination of the Greek word for tooth, *odontos*, and *Hypopomus*, type genus of Hypopomidae.

#### Brachyhypopomus (Odontohypopomus) walteri sp. n.

http://zoobank.org/81560B78-B663-4446-B0E8-C03CE4FE1279

http://species-id.net/wiki/Brachyhypopomus_walteri

**Figure 3.**
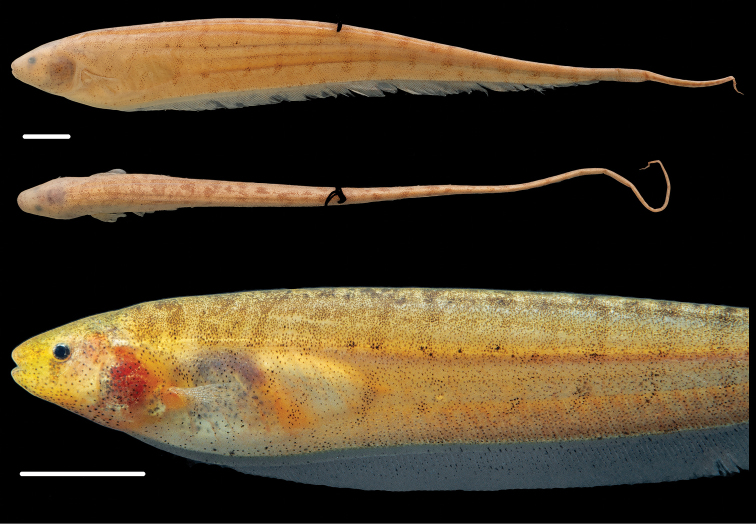
Holotype of *Brachyhypopomus walteri*, INPA 8941 (TL 163 mm, LEA 126 mm), sex undetermined, Paraná do Paracuúba, Amazonas, Brazil. Preserved whole specimen shown above close-up view of specimen immediately post-mortem. Scale bars equal 1 cm.

**Figure 4.**
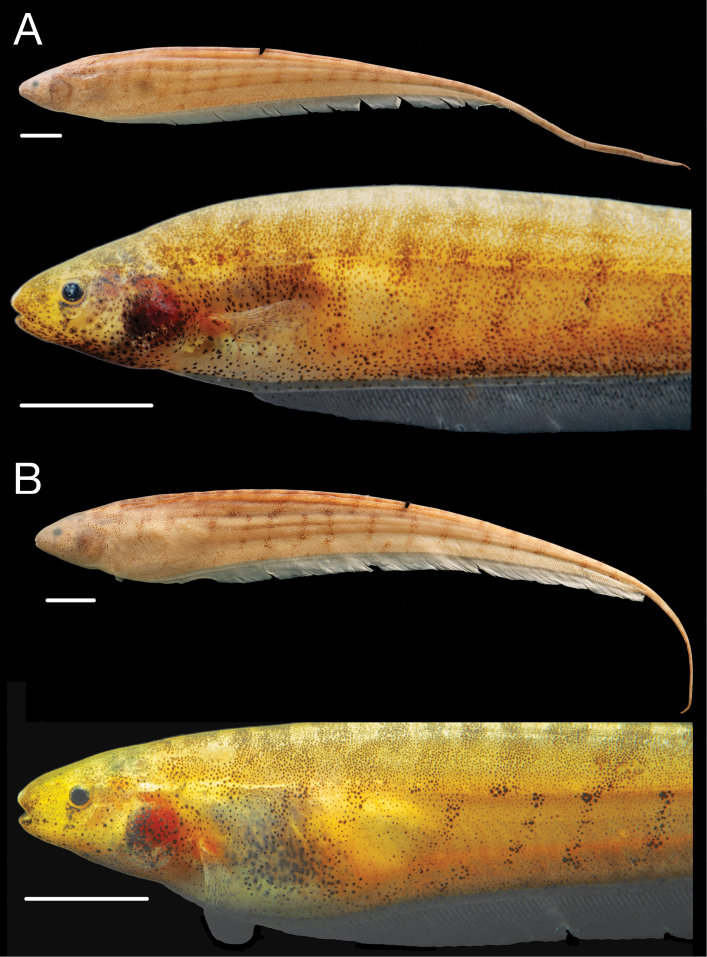
Paratypes of *Brachyhypopomus walteri*. **A** Paratype tag no. 93-55 from INPA 8880 (TL 164 mm, LEA 118 mm), sex undetermined, Ilha da Marchantaria, Rio Solimões, Amazonas, Brazil **B** Paratype tag no. 93-114 from INPA 8939 (TL 155 mm, LEA 125 mm), female, collected with holotype. Preserved whole specimens shown above close-up views of specimens immediately post-mortem. Scale bars equal 1 cm.

Figs 3 , 4 , Appendix I ;Tables 1 ,3

##### Holotype.

INPA 8941, tag no. 93-219, 163 mm TL, 126 mm LEA, sex undetermined, Amazonas, Brazil: floating meadow alongside of lake in the Paraná do Paracuúba, near mouth of Rio Negro and entrance to Lago Janauari, approx. 15 km due south of Manaus, 03°12.6'S, 059°59.4'W, J.P. Sullivan and J. Zuanon. 23 April 1993.

##### Paratypes

**(20).** Brazil: Amazonas: INPA 8926 (3 cs, tag nos. 93-58, 93-140, 93-156), collection data same as for holotype, 24 March–18 April 1993; INPA 8896 (4 alc, tag nos. 93-18, 93-19, 93-22, 93-23, 102–122 mm LEA), Ilha da Marchantaria, Rio Solimões, emergent grasses on shore of island, approx. 15 km upstream from confluence with Rio Negro, near Manaus, approx. 03°14'S, 059°59'W, J.P. Sullivan and J. Zuanon, 9 March 1993; INPA 8880 (3alc, tag nos. 93-55, 93-56, 93-57, 108–116 mm LEA), locality same as for holotype, 24 March 1993; INPA 8939 (1 alc, specimen number 93-114, 125 mm LEA), locality same as for holotype, 10 April 1993; ANSP 194031 (1 alc, tag no. JPS11-1-93/13, 84 mm LEA), channel between Rio Solimões and Lago Tefé, approx. 03°21'S, 064°40'W, J.P. Sullivan et al., 1 November 1993; ANSP 194032 (1 alc, 110 mm LEA, tag no. JPS11-20-93/1), Rio Içá in roots of water hyacinth along margin of inlet,15 km upstream of the mouth of the Içá, approx. 03°06'S, 068°05'W, J.P. Sullivan et al., 20 November 1993; ANSP 194033 (1 alc, tag no. CALH-11-20-93/2, 97 mm LEA), near Santo Antonio do Içá and mouth of Rio Içá in floating vegetation, approx. 03°07'S, 067°57'W, J.P. Sullivan et al., 20 November 1993; CUMV 97641 (1 alc, damaged, 80 mm TL, tag no. JPF-93-187/1), Rio Negro above Manaus, 03°05.38'S, 060°27.02'W, J.P. Sullivan and J.P. Friel, 14 December 1993; CUMV 97642 (5 alc, 2 damaged: tag nos. JPF-93-188/3, 188/4, 104, 100 mm LEA, 3 intact: tag nos. JPF-93-188/2, 188/5, 188/6, 95–102 mm LEA), Rio Negro above Manaus, 03°05.59'S, 60°26.83'W, J.P. Sullivan and J.P. Friel, 14 December 1993.

##### Non-types.

**Brazil**: Amazonas: Rio Solimões drainage: INHS 70542 (4 of 10, alc, 71–162 mm LEA), Ilha da Marchantaria, approx. 03°14'S, 059°59'W, P. Bayley, 14 March 1978; MZUSP 30061 (1 alc, 70 mm LEA), Rio Tefé, Lago Mucura, M. Goulding, 5 August 1979; USNM 306874 (2 alc, 91 & 100 mm LEA), Paraná da Ilha da Marchantaria, approx. 03°14'S, 059°59'W, depth 0–1.3 meters P. Bayley, 25 April 1978; USNM 306919 (1 alc, 75 mm LEA), Lago Camaleão, Ilha da Marchantaria, approx. 03°14'S, 059°59' W, P. Bayley, 29 March 1977; INPA 33268 (3 alc), Coari, 03°51.17'S, 063°28.12'W, L. Rapp Py-Daniel et al., 13 September 2013; INPA 33253 (3 alc), Manacapuru, Canaboca III, 03°35.55'S, 060°50.15'W, L. Rapp Py-Daniel et al., 17 September 2003; INPA 30241 (8 alc, 63.65–113.62 mm LEA), São Paulo de Olivença, Rio Camatiã, comunidade Monte Sinai, approx. 03°27.57'S, 068°56.00'W, L. Rapp Py-Daniel et al., 4 April 2008; ANSP 194025 (5 alc, 59.9–126.2 mm LEA, 1 cs, 117.4 mm), same data previous. Rio Uatumã: INPA 39074 (1 alc), São Sebastião do Uatumã, right bank in front of São José do Jabote, 01°56.20'S, 058°17.78'W, L. Rapp Py-Daniel et al., 1 October 2011. Rio Japurá: INPA 9945 (30 alc), Lago Caetano (várzea lake) 1 km N of Jarauá, 02°50.97'S, 064°55.70'W, W.G.R. Crampton, 8 January 1995. Rio Purus: INPA 17112 (20 alc), Paraná do Seixo, Lago Jari, 04°54.67'S, 062°21.42'W, L. Rapp Py-Daniel et al., 8 June 2001; INPA 29259 (12 alc), Beruri, Lago Ayapuá, Igarapé Ajará, 04°25.12'S, 062°15.60'W, L. Rapp Py-Daniel et al., 15 November 2007; INPA 17192 (27 alc), Igarapé das duas bocas, Paraná do Jari, 04°53.12'S, 062°20.18'W, L. Rapp Py-Daniel et al., 7 June 2001. Pará: Rio Tapajós drainage: INPA 32703 (1 alc) Rio Crepori, Jacareacanga, Igarapé do Cocho, 06°44.90'S, 056°54.72'W, W.S. Pedroza, 31 July 2008. Rio Amazonas drainage: INPA 33192 (1 alc), Almeirim, Paranaguara, 01°44.48'S, 053°10.25'W, J. Zuanon et al., 5 October 2003. Rondônia: Rio Guaporé-Madeira drainage: INPA 9721 (19 alc), 15 km above Guajará-Mirim on Rio Pacaás Novos, approx. 10°56.8'S, 065°14.3'W, G.M. dos Santos, 26 November 1983; INPA 9727 (78 alc), mouth of Rio Pacaás Novos into Rio Guaporé, G.M. dos Santos, 26 November 1983. Roraima: Rio Branco drainage: INPA 30748 (11 alc), near Boa Vista, 02°47.51’N, 060°40.15'W, L.N. Carvalho, 29 September 2006; INPA 30749 (8 alc), near Boa Vista, 02°47.51’N, 060°40.15'W, L.N. Carvalho, 29 September 2006. **Ecuador**: Río Napo drainage: FMNH 102276 (8 alc, 69–107 mm LEA), Río Napo, along edge of Lago Anangucocha, approx. 01°28.48'S, 077°33.73'W, D. Stewart et al., 13 October 1981.

##### Diagnosis.

*Brachyhypopomus (Odontohypopomus) walteri* sp. n. is diagnosed by the following three character states in combination with the character states listed above for *Odontohypopomus*: (1) patch of brown pigment below skin at base of orbit distinct; (2) body yellow and semi-translucent in living specimens; (3) caudal filament long and fine, greater than 20% of LEA in intact specimens; (4) EOD pulse duration very long (between 3.5 and 4 milliseconds at 25° C) with head-positive first phase of longer duration than second head-negative phase in both sexes (Fig. 2A, B).

In all other *Brachyhypopomus* with biphasic EODs, the second, head-negative phase of the EOD is nearly equal in amplitude or of greater duration than the head-positive first phase. *Microsternarchus bilineatus* has an EOD waveform of similar duration, but the second phase is roughly equal or longer than the first (and the repetition rate is far faster). No other species of *Brachyhypopomus* is as distinctly yellow in color, particularly in life.

This species can be distinguished from the similar *Brachyhypopomus bennetti* sp. n. by a shorter body (depth quickly tapers posteriorly: depth of body at 40^th^ post-abdominal vertebra 36–41% of depth at first abdominal vertebra vs. 46–57% in *Brachyhypopomus bennetti*), fewer anal fin rays (198–216 rays vs. 227–255 rays in *Brachyhypopomus bennetti*), a shallower electric organ, and a long, fine caudal filament (length 20–32% of LEA vs. 10–19% of LEA in *Brachyhypopomus bennetti*) with three or four bilateral columns of electrocytes at base of caudal filament (vs. six columns in *Brachyhypopomus bennetti* sp. n.). Subcutaneous pigment below eye is absent in other hypopomids and usually less conspicuous in the sister species *Brachyhypopomus bennetti* sp. n. The EOD waveform of *Brachyhypopomus walteri* sp. n. is biphasic in contrast to *Brachyhypopomus bennetti*’s monophasic EOD waveform. *Brachyhypopomus bennetti* sp. n. tends to be more darkly pigmented and less yellow and translucent.

##### Description.

Morphometric and meristic data are presented in Tables 1, 3 and 4. A *Brachyhypopomus* of moderate to small adult size for a hypopomid; largest specimen examined measures 175 mm TL, 125 mm LEA. Body very compressed, depth at posterior end of abdominal cavity 2.7–3.1 times body width. Body more compressed posteriorly, sides of body with only slight curvature posterior to abdominal cavity. Dorsal profile gently convex. Depth quickly tapers posteriorly: depth of body at 40th post-abdominal vertebra 36–41% depth at first abdominal vertebra. Head short in comparison to body length, deep and wide: HL 11.2–12.6% LEA, head depth at occiput 72–81% HL, head width at opercle 54–63% HL. Head triangular in lateral view, dorsal profile of head straight from occiput to point of downturn of snout, ventral profile of head straight from lower jaw to opercular area with little if any concavity between opercular area and tip of lower jaw. Eye moderate in size, 12.4–14.5% HL. Mouth small, terminal, jaws equal, gape 20–23% HL. Closed lips meet ventral to a horizontal through ventral margin of eye. One to five small needle–like conical teeth present on each premaxilla (Fig. 1), lower jaw edentate. Maxilla moderate in length, thin, with slight curvature. Snout moderate in length, 26–29% HL, edge of upper lip close to farthest anterior extent of snout. Posterior naris close to eye, posterior naris–eye 1.8–3.7% HL. Lateral ethmoid present. Round ossification present in anterior portion of palatine cartilage (Fig. 1). Infraorbital portion of cephalic lateralis system incomplete, lacking recurrent anterodorsal segment and associated pores beneath and anterior to the posterior nares that are present in most other *Brachyhypopomus* (see fig. 53 in Sullivan 1997); fourth supraorbital pore lying near vertical through posterior nostril, pores inconspicuous. Preopercular lateral-line canal embedded in preopercle, canals radiating out to pores. Pores of lateral-line canal immediately behind head without downward pointing tubes. Discernible lateral scales terminate along caudal filament. Five branchiostegal rays, medialmost two thin with blades oriented nearly vertically compared to outer three (see diagnosis of *Odontohypopomus*). Gill rakers robust for genus, some with weakly ossified cores, on anterior faces of first four gill arches. Rakers subtended on ceratohyals one to four by small trough-shaped ossicles. Approximately 40 gill filaments on arch one. Three pectoral radials, all partially fused together at proximal end. Mesocoracoid bridge absent. Pectoral fin broad, 12–15 branched plus unbranched rays, length 5.3–7.0% LEA. 198–216 anal fin rays, longest rays 4.0–4.9% LEA. Precaudal vertebrae 13–16, up to 75 caudal vertebrae in advance of regenerated portion of caudal filament. Body excluding head and fins covered with thin cycloid scales, small dorsally, larger posterolaterally, partially obscured by skin. Twelve scale rows above, 13 scale rows below lateral line at farthest extent of pectoral fin. Anal-fin origin slightly posterior to vertical at midpoint of extended pectoral fin. Caudal filaments long and fine in intact mature specimens, 20–32% of TL. Sexual dimorphism of caudal filaments not observed. Three or four bilateral columns of electrocytes along caudal filament, number often alternating along length of caudal filament; 38–63 rows of electrocytes. Electrocytes do not extend farther anteriorly than base of urogenital pore. No accessory electric organs on head or humeral region.

**Table 1.** Summary of morphometric measurements for *Brachyhypopomus walteri* sp. n. (included are holotype and ten paratypes identified from radiographs as undamaged at anal-fin terminus).

**Measurement**	**holotype**	**range**	**mean**	**S.D.**	**N**
Total length (TL) (mm)	163.0	116–175	153.2	15.3	11
Length to end of anal fin (LEA) (mm)	126.0	85–127	111	13	11
Head length (HL) (mm)	14.4	10.3–14.5	13	1.4	11
Caudal filament length (% TL)	22.7	19.6–31.8	27.3	3.5	11
**Proportion of LEA (%)**					
Head length	11.4	11.2–12.6	11.7	0.5	11
Snout to occiput	8.5	7.9–9.6	8.9	0.5	11
Snout to anal fin origin	19.9	18.4–21.2	19.6	0.9	11
Depth at 1st post-abdominal vertebra	12.2	10.5–13.2	12	0.8	11
Depth at 20th post-abdominal vertebra	9.4	8.5–10.0	9.4	0.5	11
Depth at 40th post-abdominal vertebra	4.7	4.4–5.2	4.7	0.3	11
Caudal filament base depth	1.9	1.6–2.1	1.8	0.2	11
Longest anal fin ray	4.6	4.0–4.9	4.5	0.2	11
Longest pectoral fin ray	5.4	5.3–7.0	6.1	0.6	11
**Proportion of HL (%)**					
Snout length	28.5	25.7–29.0	27.4	1.2	11
Gape	22.9	19.8–23.2	21.5	1.4	11
Orbital diameter	12.5	12.4–14.5	13.5	0.7	11
Interorbital distance	23.9	21.7–26.5	24	1.6	11
Posterior naris-eye	3.4	1.8–3.7	2.8	0.7	11
Branchial aperture	29.7	23.8–30.6	26.9	2.6	11
Head width at opercle	63.3	54.4–63.3	58.8	3.2	11
Head width at eye	42.2	40.1–43.8	41.9	1.3	11
Head depth at occiput	79.0	72.1–80.9	76.4	3.1	11

##### Electric organ discharge.

TheEOD is biphasic and 3.2–4.5 milliseconds in total duration at 25°C; the first head-positive phase is 1.7–1.9 times duration of second head-negative phase (Fig. 2). Resting EOD repetition rate is slow (3.1–16.3 Hz, mean 9.4 Hz, median 9.8, at 21–25°C, n=23). See Appendix II.

##### Coloration.

Background color yellow in life, yellowish-tan in preservation. In life, body semi-translucent, with gill filaments appearing cherry red through opercle, gut dark, and swim bladder whitish through abdominal wall. Pigmentation variable: poorly to moderately developed irregular bands along sides, darker and wide above lateral line, often with a spot of darker intensity on lateral line itself. Bands either restricted to anterior portion of body above lateral line or connected to fainter bands below. Some bands connect to eight to 12 irregular saddles across dorsum. Saddles more regular in smaller individuals. Dorsal rami of the anterior lateral line nerve visible when viewed from above as two thin, dark parallel lines running along upper back beginning a short distance behind head and continuing to mid-point of the back. Cheeks, underside of head and sides of body below lateral line peppered with prominent dark brown stellate chromatophores that greatly contrast with background color of skin and that do not form part of a larger pattern. Diffuse pigment below eye resembling a teardrop is more prominent in live specimens as overlying tissue becomes opaque upon preservation. Pectoral and anal fin with irregular brown pigment along rays; interradial membranes hyaline.

##### Distribution and ecology.

**Figure 5.**
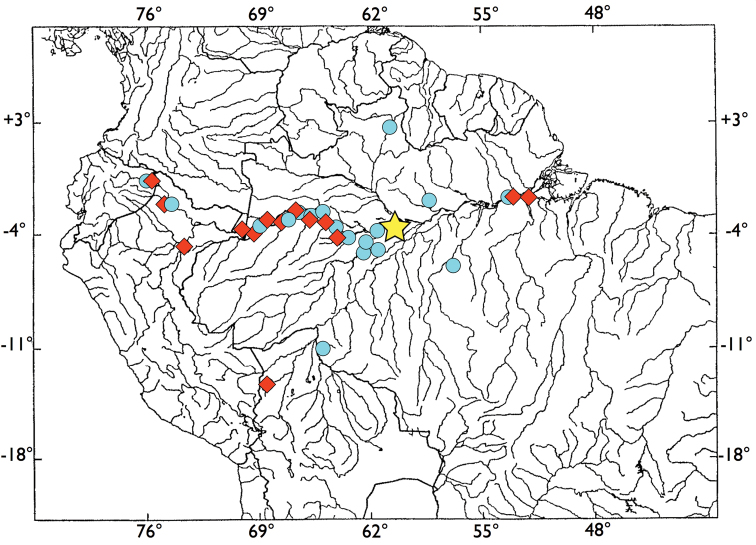
Distribution of examined specimens of *Brachyhypopomus walteri* sp. n. (blue circles) and *Brachyhypopomus bennetti* sp. n. (red diamonds). Common holotype locality for both species indicated by yellow star.

See distribution map (Fig. 5). *Brachyhypopomus walteri* sp. n. is known only from the Amazon basin where it appears to be common in floating meadow habitats, (mostly composed of the grass *Paspalum repens*, Poaceae), on the margins of the Amazonas/Solimões and its tributaries. It has been collected predominantly in white water, but also in areas near the confluence of black water rivers with the Amazonas/Solimões ranging from low to medium conductivity. Apart from one collection very near Manaus and the white water Rio Branco, it is absent from collections in the Rio Negro system. It is frequently taken with *Brachyhypopomus bennetti* sp. n. and sometimes with *Brachyhypopomus brevirostris*. Species of *Eigenmannia*, *Gymnotus*, the apteronotid *Parapteronotus hasemani* and the electric eel, *Electrophorus electricus*, frequently co-occur in the floating meadow habitats preferred by this species.

##### Etymology.

This species is named for Walter Heiligenberg (1938–1994) in honor of his discoveries in electric fish neurophysiology and behavior made at the Scripps Institute of Oceanography. These notably include the “jamming avoidance response” in *Eigenmannia*, often described as the best-understood vertebrate behavior.

#### Brachyhypopomus (Odontohypopomus) bennetti sp. n.

http://zoobank.org/20BDF5D2-49FD-4B21-819B-54C4684B9772

http://species-id.net/wiki/Brachyhypopomus_bennetti

**Figure 6.**
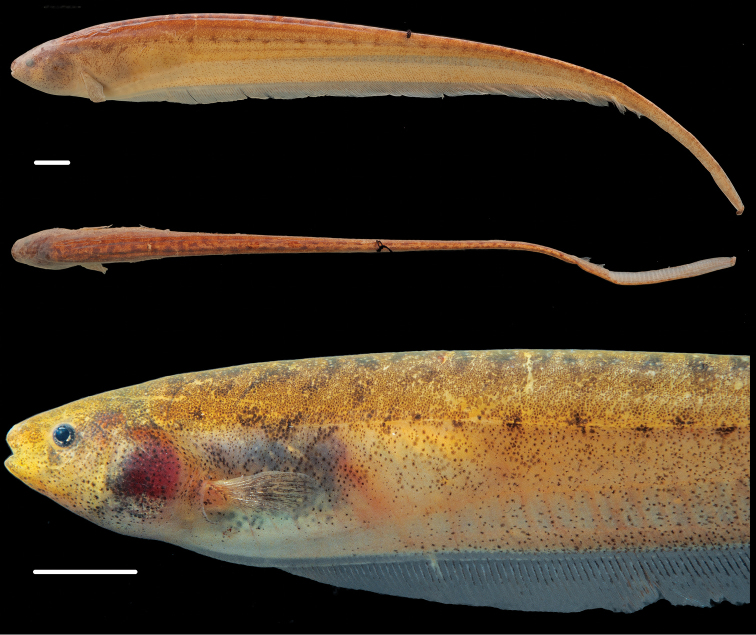
Holotype of *Brachyhypopomus bennetti*, INPA 39560 (TL 215 mm, LEA 171 mm), female, Paraná do Paracuúba, Amazonas, Brazil. Preserved whole specimen shown above close-up view of specimen immediately post-mortem. Scale bars equals 1 cm.

**Figure 7.**
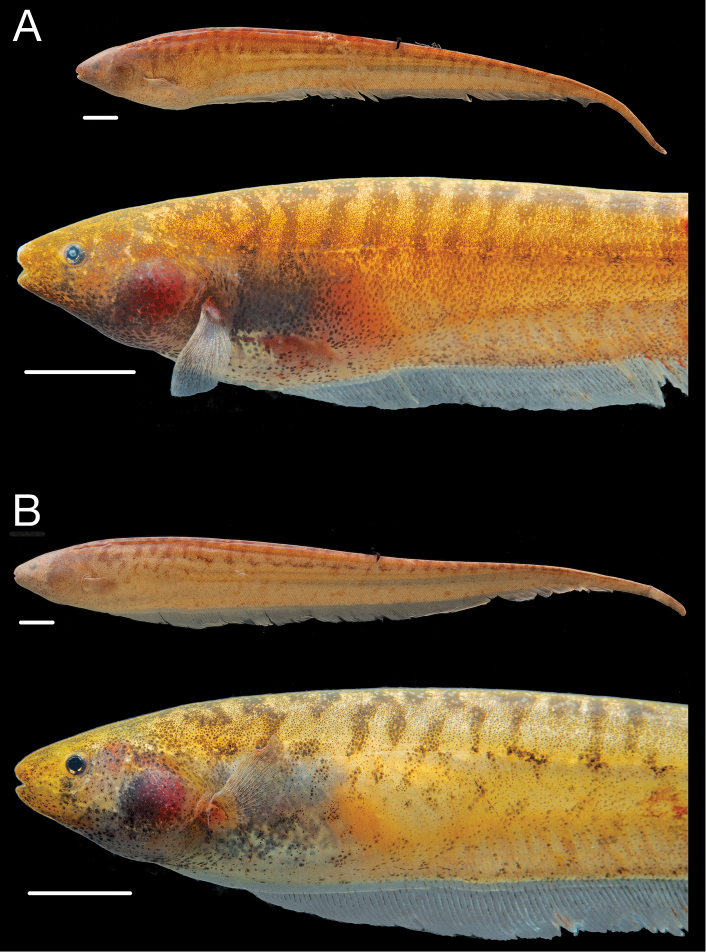
Paratypes of *Brachyhypopomus bennetti*. **A** Paratype tag no. 93-214 from INPA 39581 (TL 175 mm, LEA 150 mm), female, Lago Janauari, Amazonas, Brazil **B** Paratype tag no. 93-137, INPA 8940 (TL 190 mm, LEA 167 mm), Lago Janauari. Preserved whole specimens shown above close-up views of specimens immediately post-mortem. Scale bars equal 1 cm.

Figs 6 , 7 , Appendix I ; Tables 2 , 3

##### Holotype.

INPA 39560 (ex-8941), tag no. 93-220, 215 mm TL, 176 mm LEA, female, Amazonas, Brazil: floating meadow along side of lake in the Paraná do Paracuúba, near mouth of Rio Negro and entrance to Lago Janauari, approx. 15 km due south of Manaus, 03°12.6'S, 059°59.40'W, J.P. Sullivan and J. Zuanon, 23 April 1993.

##### Paratypes

**(18).** Brazil: Amazonas: INPA 39561 (1 cs, tag no. 93-221, 95 mm LEA, damaged), same data as holotype; INPA 39579 (1 alc, tag no. 93-26, 152 mm LEA, damaged), Ilha da Marchantaria, approx. 03°14'S, 059°59'W, J.P. Sullivan & J. Zuanon, 5 March 1993; INPA 8862 (2 alc, tag nos. 93-139, 93-163, 152 mm & 162 mm LEA, respectively, damaged), Paraná do Paracuúba near holotype locality, J.P. Sullivan & J. Zuanon, 14 April 1993; INPA 8863 (1 alc, tag no. 93-222, 145 mm LEA, damaged), Lago Janauari & Paraná do Paracuúba near holotype locality, J.P. Sullivan & J. Zuanon, 24 April 1993; INPA 39578 (1 alc, tag no. 93-54, 153 mm LEA, damaged), Paraná do Paracuúba near holotype locality, J.P. Sullivan & J. Zuanon, 24 March 1993; INPA 39580 (3 alc, 2 damaged: tag nos. 93-112, 93-113, 160 &170 mm LEA respectively, 1 intact: tag no. 93-116, 161 mm LEA), same data as for holotype; INPA 8940 (3 alc, all intact, tag nos. 93-137, 93-141, 93-165, 167–171 mm LEA), lake between Janauari and Solimões, approx. 03°12.6'S, 060°01.9'W, J.P. Sullivan & J. Zuanon, 14 April 1993; INPA 39581 (1 alc, tag no. 93-214, 150 mm LEA, intact), same data as previous; ANSP 194034 (1 alc, tag no. JPS-93-44/1, 125 mm LEA, intact), floating grasses directly in front of town of Fonte Boa, 02°30.57'S, 066°05.72'W, J.P. Sullivan, 11 November 1993; ANSP 194035 (1 alc, tag no. JPS-93-57/1, 89 mm LEA, intact), Rio Jutaí near mouth into Solimões, floating grasses and water hyacinth, approx. 02°40'S, 066°40'W, J.P. Sullivan, 16 November 1993; CUMV 97640 (3 alc, 1 intact: tag no. JPS-93-37/1, 98 mm LEA, 2 damaged: tag nos. JPS-93-37/2, JPS-93-37/3, 118 & 75 mm LEA, respectively), Rio Juruá near mouth into Solimões, approx. 02°40'S, 065°45'W, floating vegetation, J.P. Sullivan, 8 November 1993.

##### Non-types.

**Bolivia**: Beni: Río Beni drainage: CAS 72216 (1 alc, 126 mm LEA), Reyes, 24 mi. NE of Rurrenabaque on the pampas, approx. 14°17'S, 067°20’ W, N.E. Pearson, Mulford Expedition, 15 November 1921; CAS 81631 (1 alc, size n.a.), data same as for previous. **Brazil**: Amazonas: Rio Solimões drainage: INHS 70542 (5 of 10, alc, 71–162 mm LEA), Ilha da Marchantaria south of Manaus, approx. 03°14'S, 059°58’ W, P. Bayley, 14 March 1978; MCZ 78163 (1 of 8, alc, 98 mm LEA,) Lago Jacaretinga near Manaus, T.J. Zaret et al., 5 November 1979; USNM 306841 (1 alc, 147 mm LEA), Paraná do Lago Januauacá, entrance to Lago do Castanho, 0–2.1 meters depth, P. Bayley, 30 March 1977; USNM 306859 (1 alc, 72 mm LEA), Lago Terra Preta, Janauari, depth 0–1 meter, P. Bayley, 3 August 1977; USNM 306875 (1 alc, 131 mm LEA), Lago Janauari, near its outflow, depth 0.6–1 meters; USNM 306929 (10 alc, 71–139 mm LEA), Lago Camaleão, near Manaus within Ilha da Marchantaria, P. Bayley, 28 March 1977; USNM 306947 (1 alc, 96 mm LEA), collection locality same as for previous, P. Bayley, 29 March 1977; INPA 13569 (2 alc), Iranduba, Ilha da Marchantaria, Lago Camaleão, P. Petry & R. Sotero, 3 April 1993; INPA 33255 (3 alc), Manaus, Ilha da Paciência, 03°16.68'S, 060°16.58'W, L. Rapp Py-Daniel et al., 18 September 2003; INPA 32091 (3 alc), Manaus, Lago do Rei, J.I.S. Botero, 30 June 1998; INPA 6403 (5 alc), Iranduba, Lago Janauacá, INPA staff, 12 September 1979; INPA 39563 (4 alc), Coari, 03°51.17'S, 063°28.12'W, L. Rapp Py-Daniel et al., 13 September 2003; INPA 33270 (3 alc), Fonte Boa, Lago “Ressaca Grande”, 02°28.43'S, 066°09.28'W, J. Zuanon et al., 8 September 2003; INPA 39562 (5 alc, 54.9-144.1 LEA), São Paulo de Olivença, Rio Camatiã, comunidade Monte Sinai, approx. 03°27.57'S, 068°56.00'W, L. Rapp Py-Daniel et al., 4 April 2008; ANSP 194026 (4 alc, 65.1–132.6 mm LEA), same data as previous; INPA 18244 (6 alc) Alvarães, Lago Geraldo, Reserva Mamirauá, W.G.R. Crampton, 29 May 1998; INPA 18245 (4 alc), Alvarães, Lago Curuçá Aberto, Reserva Mamirauá, W.G.R. Crampton, 1 June 1998; INPA 18246 (3 alc), Alvarães, Lago Curúça Comprido, Reserva Mamirauá, W.G.R. Crampton, 2 June 1998; INPA 15816 (34), INPA 18241 (1 alc), INPA 18242 (1 alc), INPA 18243 (1 alc), INPA 18348 (1 alc), INPA 18349 (1 alc), INPA 18350 (1 alc), 18351 (2 alc), INPA 18352 (1 alc), INPA 18354 (3 alc), Alvarães, Lago Mamirauá system, W.G.R. Crampton, 1 April 1997–18 May 1998; INPA 18355 (3 alc), Alvarães, Lago Mamirauá, Lago Promessa, W.G.R. Crampton, 19 May 1998; INPA 18357 (3 alc), Alvarães, Lago Curuçá, Lago Mamirauá system, W.G.R. Crampton, 30 May 1998; INPA 18358 (2 alc), Alvarães, Lago Miratinin, Lago Mamirauá system, W.G.R. Crampton, 4 June 1998; INPA 18361 (2 alc), Lago Apolônio, Lago Mamirauá system, W.G.R. Crampton, 1 July 1999; INPA 18362 (1 alc), Lago Secretária, Lago Mamirauá system, W.G.R. Crampton, 9 June 2000; INPA 33196 (1 alc), Tabatinga, Comunidade Palmares, 03°57.89'S, 069°20.19'W, J. Zuanon et al., 2 September 2003. Rio Jutaí drainage: INPA 33187 (3 alc), Ressaca do Luizinho, 02°42.98'S, 066°48.22'W, J. Zuanon et al., 6 September 2003. Pará: Rio Amazonas drainage: INPA 39564 (1 alc), Almeirim, Comunidade Paranaguara, 01°44.48'S, 053°10.25'W, J. Zuanon et al., 5 October 2003. Rio Tapajós drainage: INPA 39518 (1 alc), Belterra, Igarapé do Índio near mouth to Rio Tapajós, 02°40'S, 054°58'W, F.R. Ribeiro, 26 December 2008. **Colombia**: Amazonas: Río Amazonas drainage: FMNH 85363 (38 alc, 67–153 mm LEA), Río Amazonas 2–3 miles upstream of Leticia, approx. 04°05'S, 070°03'W, Navarro, Thomerson et al., 13 November 1973; USNM 216870 (1 alc, 110 mm LEA), Leticia, D. Kramer, 4 December 1974. **Ecuador**: Napo: Río Napo drainage: FMNH 102270 (2 alc, 56 & 110 mm LEA), Laguna de Limoncocha, D. Stewart et al., 4 October 1981. **Peru**: Loreto: Río Ucayali drainage: AMNH 78060 (7 of 14, alc, 81–101 mm LEA), several sites along 10 km stretch, Ferraris, Montrevil et al., 7 July 1987.

##### Diagnosis.

**Figure 8.**
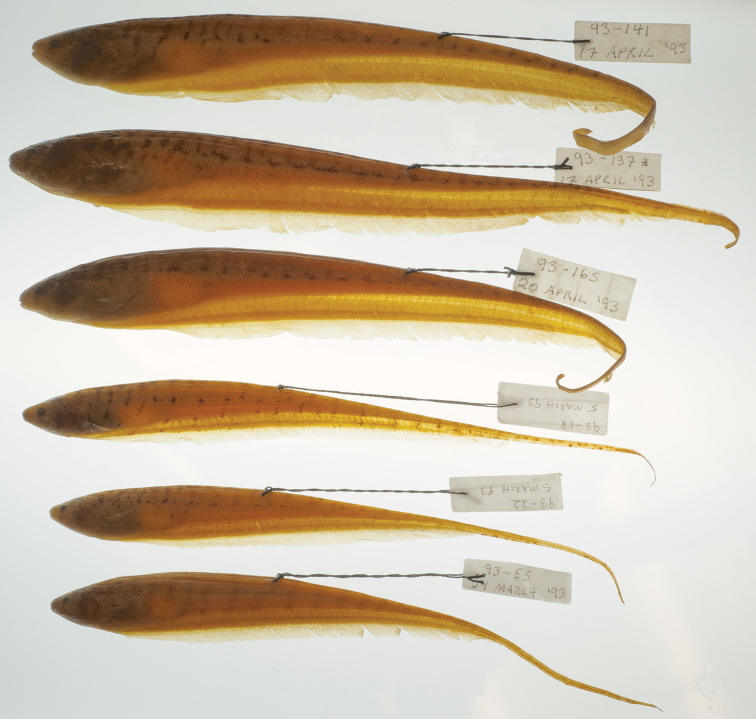
Paratypes of *Brachyhypopomus bennetti* sp. n. (top 3) and *Brachyhypopomus walteri* sp. n. (bottom 3) showing transparency of electric organ to transmitted light and comparative depth of the electric organ in the two species.

*Brachyhypopomus (Odontohypopomus) bennetti* sp. n. is diagnosed by two character states in addition to those used to diagnose the subgenus *Odontohypopomus* above: (1) electric organ along caudal filament and along body above anal fin exceedingly deep and visible as large semi-translucent area, occupying approximately 14–17% body depth directly posterior to abdominal cavity; (2) monophasic, head-positive EOD, approximately 2.1 milliseconds in duration in both sexes at 25°C. The appearance of the electric organ in this species when backlit (Fig. 8) is significantly larger than in any other species of *Brachyhypopomus*. No other described *Brachyhypopomus* has a monophasic EOD waveform.

This species can be distinguished from the similar *Brachyhypopomus walteri* sp. n. by a longer body (depth gradually tapers posteriorly: depth of body at 40^th^ post-abdominal vertebra 46–57% vs. 36–41% of depth at first abdominal vertebra vs. in *Brachyhypopomus walteri*), more numerous anal fin rays (227–255 vs. 198–216 in *Brachyhypopomus walteri*), a deeper electric organ along the body and a short, deep caudal filament (10–19% of LEA vs. 20–32% of LEA in *Brachyhypopomus bennetti*) with six bilateral columns of electrocytes at its base (vs. three or four columns in *Brachyhypopomus bennetti* sp. n.). Subcutaneous pigment suggestive of a teardrop below eye is usually less conspicuous than in the sister species *Brachyhypopomus walteri* sp. n., although often present. The EOD waveform of *B bennetti* sp. n. is monophasic in contrast to *Brachyhypopomus walteri*’s biphasic EOD waveform. *Brachyhypopomus walteri* sp. n. tends to be less darkly pigmented and more translucent and yellowish in life than *Brachyhypopomus bennetti* sp. n.

##### Description.

Morphometric and meristic data are presented in Tables 2–4. A *Brachyhypopomus* of moderate adult size for a hypopomid; largest specimen examined measures 232 mm TL, 189 mm LEA. Body very long and compressed, depth at posterior end of abdominal cavity 2.6 to 2.9 times body width. Body more compressed posteriorly, but sides of body with only slight curvature posterior to abdominal cavity. Dorsal profile gently convex. Depth gradually tapers posteriorly: depth of body at 40th post-abdominal vertebra 46-57% of depth at first abdominal vertebra. Head short in comparison to body length, deep and wide: HL 10.3–12.3% LEA, head depth at occiput 76–80% HL, head width at opercle 58–65% HL. Head triangular in lateral view: dorsal profile of head straight from occiput to point of downturn of snout, ventral profile of head straight from lower jaw to opercular area with little if any concavity between opercular area and tip of lower jaw. Eye moderate in size, 11–14% HL. Mouth small, terminal, jaws equal, gape 21–26% HL. Closed lips meet ventral to a horizontal through ventral margin of eye. One or more small needle-like conical teeth present on each premaxilla. This feature is variable, in one case observed only unilaterally. Lower jaw edentate. Maxilla moderate in length, thin, with slight curvature. Snout length moderate, 26–30% HL, edge of upper lip close to farthest anterior extent of snout. Posterior nostril particularly small and close to eye: posterior naris-eye 2.3–4.3% HL. Lateral ethmoids present. Round ossification present in anterior of palatine cartilage. Infraorbital portion of cephalic lateralis system incomplete, lacking recurrent anterodorsal segment and associated pores beneath and anterior to the posterior nares present in most other *Brachyhypopomus* (see fig. 53 in Sullivan 1997); fourth supraorbital pore lying near vertical through posterior nostril, pores inconspicuous. Preopercular lateral-line canal embedded in preopercle, canals radiating out to pores. Pores of lateral-line canal immediately behind head without downward pointing tubes. Discernible lateral scales terminate along caudal filament. Five branchiostegal rays, medialmost two thin compared to outer three, blades oriented nearly vertically. Gill rakers robust for genus, some with weakly ossified cores, on anterior faces of first four gill arches. Rakers subtended on ceratohyals 1–4 by small trough-shaped ossicles. Approximately 50 gill filaments on arch one. Three pectoral radials, with partial fusion of all three at proximal end. Mesocoracoid bridge absent. Pectoral fin broad, robust, 14–17 branched plus unbranched rays, length 5.4–7.5% LEA. Anal-fin rays 227–255, length 4.0–4.5% LEA. Precaudal vertebrae 13–15, a high proportion of specimens show signs of damage and regeneration above anal fin terminus. As many as 72 caudal vertebrae in advance of regenerated portion of caudal filament. Body excluding head and fins covered with thin cycloid scales, small dorsally, larger posterolaterally, partially obscured by skin. Twelve scale rows above, 16 scale rows below lateral line at farthest extent of pectoral fin. Anal-fin origin near vertical through midpoint of extended pectoral fin. Caudal filaments short in intact mature specimens: 10–19% TL. Sexual dimorphism of caudal filaments not noted. Six columns of electrocytes at base of caudal filament, number of columns reduce to three or two along length of filament; 22–35 rows of electrocytes. Electrocytes do not extend anterior to base of urogenital pore. No accessory electric organs on head or humeral region.

**Table 2.** Summary of morphometric measurements for *Brachyhypopomus bennetti* sp. n. (included are holotype and nine paratypes, identified from radiographs as undamaged at anal-fin terminus).

**Measurement**	**holotype**	**range**	**mean**	**S.D.**	**N**
Total length (TL) (mm)	215.0	98–215	173	44.2	10
Length to end of anal fin (LEA) (mm)	176.0	89–176	149.9	32.4	10
Head length (HL) (mm)	18.5	10.8–19.6	16.7	3.2	10
Caudal filament length (% TL)	18.1	1.4–18.9	12.1	5.6	10
**Proportion of LEA (%)**					
Head length	10.5	10.3–12.3	11.2	0.7	10
Snout to occiput	8.2	7.7–9.9	8.6	0.7	10
Snout to anal fin origin	17.0	16.7–19.0	17.5	0.8	10
Depth at 1st post-abdominal vertebra	10.8	10.6–12.9	11.6	0.7	10
Depth at 20th post-abdominal vertebra	8.7	8.5–10.1	9.1	0.5	10
Depth at 40th post-abdominal vertebra	5.3	5.1–6.2	5.6	0.3	10
Caudal filament base depth	2.4	1.3–3.2	2.1	0.6	10
Longest anal fin ray	4.0	4.0–4.5	4.3	0.2	10
Longest pectoral fin ray	5.7	5.4–7.5	6.0	0.7	10
**Proportion of HL (%)**					
Snout length	27.7	25.7–29.5	27.6	1.3	10
Gape	21.8	21.2–25.5	22.6	1.6	10
Orbital diameter	13.4	11.6–14.1	12.7	0.9	10
Interorbital distance	28.4	22.4–28.4	25.1	1.9	10
Posterior naris-eye	0.4	2.3–4.3	3.3	0.7	10
Branchial aperture	29.3	26.4–33.6	30.3	2	10
Head width at opercle	64.2	58.4–64.8	62.0	2.1	10
Head width at eye	44.9	39.9–44.9	42.4	1.7	10
Head depth at occiput	80.3	76.3–80.3	78.3	1.5	10

**Table 3.** Anal-fin ray counts of *Brachyhypopomus walteri* sp. n. and *Brachyhypopomus bennetti* sp. n. (holotypes and paratypes) compared to seven other *Brachyhypopomus* species; modal values **highlighted**.

**Species**	**170–179**	**180–189**	**190–199**	**200–209**	**210–219**	**220–229**	**230–239**	**240–249**	**250–259**	**260–269**	**270–279**
***Brachyhypopomus walteri* sp. n.**			2	**5**	2						
***Brachyhypopomus bennetti* sp. n.**						1	**4**	3	2		
*Brachyhypopomus brevirostris*							2	**5**	4	3	2
*Brachyhypopomus bullocki*			4	5	**13**	4	3				
*Brachyhypopomus beebei*					4	**5**	2				
*Brachyhypopomus pinnicaudatus*	2	**5**	2	2	1						
*Brachyhypopomus occidentalis* (types)				**2**							
*Brachyhypopomus occidentalis* (Maracaibo Basin)			2	**6**	2						
*Brachyhypopomus diazi*			3	**16**	9	4					
*Brachyhypopomus janeiroensis*			3	**7**							

**Table 4.** Precaudal vertebrae counts of *Brachyhypopomus walteri* sp. n. and *Brachyhypopomus bennetti* sp. n. (holotypes and paratypes) compared to seven other *Brachyhypopomus* species; modal values **highlighted**.

**Species**	**11**	**12**	**13**	**14**	**15**	**16**	**17**	**18**
***Brachyhypopomus walteri* sp. n.**			4	**5**	1	1		
***Brachyhypopomus bennetti* sp. n.**			2	**5**	3			
*Brachyhypopomus brevirostris*				5	**11**	4		
*Brachyhypopomus bullocki*	2	21	6					
*Brachyhypopomus beebei*							**11**	4
*Brachyhypopomus pinnicaudatus*				2	13	**20**	1	
*Brachyhypopomus occidentalis* (types)				1	**4**	2		
*Brachyhypopomus cf. occidentalis* (Maracaibo Basin)			1	**8**	1			
*Brachyhypopomus diazi*			7	**31**	2			
*Brachyhypopomus janeiroensis*					2	**8**		

##### Electric organ discharge.

The EOD has a simple, head-positive monophasic waveform with a total duration 1.9–2.4 milliseconds at 25°C (Fig. 2). No sexual dimorphism has been observed. Resting EOD rate is very slow (2.0–8.9 Hz, mean 4.7 Hz, median 4.9 Hz, at 21–25° C, n=31). See Appendix III.

##### Coloration.

Background color yellowish-tan in life, brownish-tan in preservation. Pigmentation variable: poorly to moderately developed irregular bands along sides, darker and wide above lateral line, often with a spot of darker intensity on lateral line itself. Bands either restricted to anterior portion of body above lateral line or connected to fainter bands below. Some bands connect to 8–12 irregular saddles across dorsum. Saddles more regular in smaller individuals. Dorsal rami of the anterior lateral line nerve visible when viewed from above as two thin, dark parallel lines running along upper back beginning a short distance behind head and continuing to mid-point of the back. Cheeks, underside of head and sides of body below lateral line peppered with prominent dark brown stellate chromatophores that greatly contrast with background color of skin and that do not form part of a larger pattern. Pectoral and anal fins with irregular brown pigment along rays, interradial membranes hyaline.

##### Distribution and ecology.

See distribution map, Fig. 5. *Brachyhypopomus bennetti* sp. n. is known only from Amazon Basin where it appears to be common in floating meadow habitats on the margins of the Amazonas/Solimões River and its tributaries. Its distribution and habitat preference seems very similar to that of its sister species, *Brachyhypopomus walteri* sp. n., with which it is often collected.

##### Etymology.

This species is named for Michael V.L. Bennett of the Albert Einstein College of Medicine of Yeshiva University, Bronx, New York, in honor of his pioneering work on electric fish neurophysiology. Bennett (1961, 1971) reported studying a *Brachyhypopomus* (therein *Hypopomus*) with a monophasic EOD likely to have been this species.

### Key to the species of subgenus *Odontohypopomus*

**Table:** 

1	Anal rays 198–216, caudal filament 20–32% LEA (in intact individuals), electric organ not particularly deep, with three or four electrocyte columns at origin of caudal filament, subcutaneous pigment below eye prominent, EOD biphasic, 3.2–4.5 milliseconds in total duration	*Brachyhypopomus walteri* sp. n.
2	Anal rays 227–255, caudal filament 10–19% LEA (in intact individuals), electric organ deep, with six electrocyte columns at origin of caudal filament, subcutaneous pigment below eye present or absent, EOD monophasic, 1.9–2.4 milliseconds in duration	*Brachyhypopomus bennetti* sp. n.

## Discussion

### Affinities of the new subgenus

Together, several shared character states unobserved in other Hypopomidae and so presumably derived (teeth on the premaxillae, branchiostegal-ray orientation, similar pigmentation and long duration EODs with slow repetition rates) are strong evidence that these two new species are closest relatives among the described *Brachyhypopomus*. An unpublished analysis of mitochondrial DNA sequences in Sullivan (1997) further supports this conclusion. We take the step of erecting the new subgenus *Odontohypopomus* to provide a name to this distinctive, toothed subgroup of *Brachyhypopomus* that will serve to unite them should the genusbe reorganized in the future. Other subgroups of *Brachyhypopomus* have already been recognized, although this one is the first to be formerly named. (Until others are, all other *Brachyhypopomus* species belong to the nominotypical subgenus *Brachyhypopomus* by default.) While phylogenetic relationships among all the described species of hypopomids remain to be determined, the available morphological and molecular data in Sullivan (1997) in combination with the subsequent descriptions of *Brachyhypopomus* species suggest the following natural subgroups withing the genus: (1) the “*beebei*-group” (unnamed Clade E in Sullivan 1997): including *Brachyhypopomus beebei*, *Brachyhypopomus pinnicaudatus*, *Brachyhypopomus gauderio*, and *Brachyhypopomus draco*; (2) the “*occidentalis* group” (unnamed Clade F in Sullivan 1997) including *Brachyhypopomus occidentalis*, and *Brachyhypopomus diazi*; and (3) the “*brevirostris* group” (unnamed Clade B in Sullivan 1997) including *Brachyhypopomus brevirostris* and *Brachyhypopomus bullocki*. Affinities of other species to these groups are less certain. *Brachyhypopomus janeiroensis* Costa and Campos da Paz, 1992shares at least one derived condition with *Brachyhypopomus beebei* and *Brachyhypopomus pinnicaudatus*: a free preopercular lateralis canal (Sullivan 1997). From its description and photo, *Brachyhypopomus jureiae* Triques & Khamis, 2003 would appear to be a close relative of *Brachyhypopomus janeiroensis* while the position of *Brachyhypopomus bombilla* Loureiro & Silva, 2006 within *Brachyhypopomus* is difficult to assess from its description alone. We leave the task of recognizing additional subgenera of *Brachyhypopomus* to other authors currently revising the group.

### Distinguishing between *Brachyhypopomus walteri* sp. n. and *Brachyhypopomus bennetti* sp. n.

When first collected, we considered whether these two morphotypes represented sexual dimorphism within a single species (indeed the two are frequently lumped in existing museum lots), but the observation of unambiguous males and females within each type dispelled this possibility. The two species are most easily distinguished from each other by differences in the thickness of the electric organ, the length of the caudal filament and their EOD, as well as by anal-fin ray counts. *Brachyhypopomus bennetti* sp. n. has a short but very deep caudal filament (length less than 20% TL) and a monophasic EOD waveform. By contrast, caudal filaments of *Brachyhypopomus walteri* sp. n. measure 20–30% TL in individuals with sufficiently regenerated caudal filaments and this species has a very long, biphasic EOD waveform. Damage to the caudal portion of the body is common in both species and many specimens show incomplete regeneration. The electric organ of *Brachyhypopomus bennetti* has six columns of electrocytes at the base of the caudal filament, that of *Brachyhypopomus walteri* only three or four, and *Brachyhypopomus bennetti*’s organ viewed with transmitted light appears to occupy much more tissue above the anal fin musculature, along length of the body, than in *Brachyhypopomus walteri* (Fig. 8). The EODs of *Brachyhypopomus bennetti* are monophasic, while those of *Brachyhypopomus walteri* are biphasic (Fig. 2). *Brachyhypopomus bennetti* have 227–255 anal fin rays while *Brachyhypopomus walteri* have considerably fewer, 198–216. Furthermore, the teardrop-like pigment below the eye is usually more prominent in *Brachyhypopomus walteri*, although also present to varying degrees in individuals of both species. We have examined an insufficient sample of cleared and stained specimens to judge if there are differences in the number of premaxillary teeth between the species, although in a sample of three cleared and stained specimens we found no more than two teeth per premaxilla in *Brachyhypopomus bennetti* while up to five teeth per premaxilla in a sample of four cleared and stained *Brachyhypopomus walteri*. In both species, the number of teeth is variable. Unfortunately, it is difficult to see these teeth in alcoholic specimens as they are obscured by thick, opaque tissue lining the roof of the mouth.

### Ecological and evolutionary considerations

**Figure 9.**
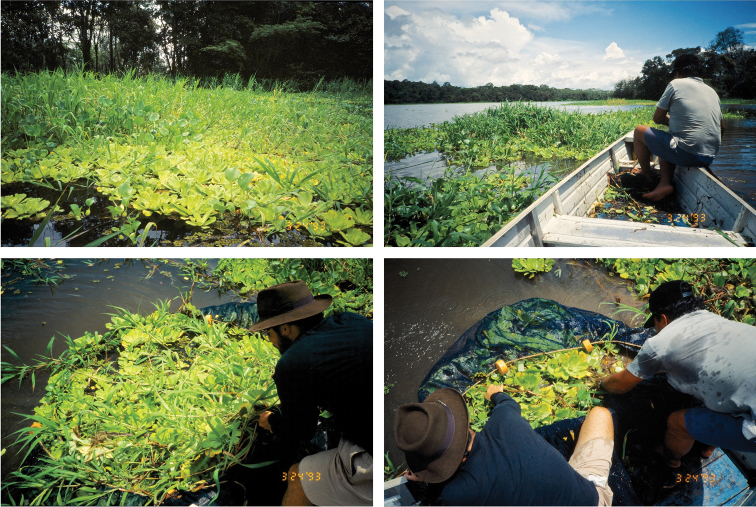
Type locality and habitat for *Brachyhypopomus bennetti* sp. n. and *Brachyhypopomus walteri* sp. n. and method of capture with seines pulled underneath floating vegetation (*Paspalum repens* and *Eichhornia crassipes*) from a motorboat. Paraná do Paracuúba, south of Manaus, Brazil, approximately 03°12.6'S, 59°59.4'W.

The probable sister-species status of *Brachyhypopomus walteri* and *Brachyhypopomus bennetti* is especially interesting given that they are frequently collected together (including at their common type locality) and have a broadly overlapping geographic distribution. Both species are known exclusively from the Amazonas/Solimões basin and seem to prefer the root tangle of large, floating grass meadows that are common along the margins of the Amazon’s river-floodplain system (Fig. 9). Why the ancestor of these two species would have regained oral teeth remains an interesting question, one that could perhaps be addressed by future studies of the diet and feeding behavior of these two species relative to their congeners.

*Brachyhypopomus bennetti* is unusual both for its remarkably large electric organ (as proportion of body depth occupied, depth of the caudal filament and a high number of horizontal columns of electrocytes) and an unusual EOD waveform that consists of a simple, head-positive pulse of long duration. Hopkins (1999) suggested that the differences in electric organ structure among species of *Brachyhypopomus* may often be adaptations to the conductivity of water in a species’ preferred environment. Other species with five or six parallel columns of electrocytes and short caudal filaments such as *Brachyhypopomus diazi* and *Brachyhypopomus occidentalis* tend to be found in high conductivity environments (above 150 µS/cm), while species with three columns and extended caudal filaments such as *Brachyhypopomus brevirostris* and *Brachyhypopomus bullocki* are most commonly found in lower conductivity environments (often well below 100 µS/cm). The first type of organ, with more electrocytes in parallel, but fewer in series, has low internal resistance and is adapted to generating current in water with low resistivity (high conductivity). The latter type, with more electrocytes in series, but fewer in parallel, has higher internal resistance and is capable of generating the higher voltages necessary for passing current through highly resistive (low conductivity) water. Thus, species-specific differences in electric organ structure may often reflect impedance-matching to water conductivity regimes (Hopkins 1999).

Conductivity in the white water floating meadow habitat where *Brachyhypopomus bennetti* and *Brachyhypopomus walteri* were collected is intermediate for Neotropical freshwater habitats: between 60 and 100 µS/cm (pers. obs.). The characteristics of *Brachyhypopomus walteri*’s electric organ (3 or 4 columns, intermediate length caudal filament) are similar to those seen in the probable sister clade to *Odontohypopomus*, *Brachyhypopomus pinnicaudatus* + *Brachyhypopomus beebei*. (Although these latter two species similarly occupyintermediate resistivity environments and are sometimes collected within floating meadows, they are primarily found farther from large river channels in “terra firme” creeks and lagoons.) Given this phylogenetic assumption, it seems probable that *Brachyhypopomus bennetti*’s enlarged electric organ with five or six horizontal electrocyte columns evolved from an ancestor with an electric organ similar to that seen in *Brachyhypopomus walteri*, *Brachyhypopomus pinnicaudatus* and *Brachyhypopomus beebei*, but as an adaptation to something other than high water conductivity.

In a study that considered selective pressures on gymnotiform EOD waveform evolution, and in which *Brachyhypopomus bennetti* and *Brachyhypopomus walteri* were identified as “sp. 1” and “sp. 2,” respectively, Stoddard (1999) reported that amplitude-calibrated recordings of *Brachyhypopomus bennetti*’s monophasic EOD show them to be very much more powerful than those of other, similarly sized *Brachyhypopomus* species and between five and ten times greater amplitude than those of *Brachyhypopomus walteri*. The unusual EOD waveform, hypertrophied electric organ and high-amplitude EOD of *Brachyhypopomus bennetti* invites inquiry into the possible adaptive value of these features.

Biphasic EODs in pulse gymnotiforms may have evolved from primitive monophasic EODs as a means to reduce the low frequency/direct current component of the signal to which electroreceptive predators (other gymnotiforms and catfishes equipped with ampullary-type electroreceptors) are sensitive (Stoddard 1999, Stoddard 2002, Stoddard and Markham 2008). Monophasic EODs are rare among modern adult gymnotiforms with pulse-type EODs: the only species reported to have them apart from *Brachyhypopomus bennetti*, *Electrophorus electricus* (itself an electroreceptive predator) and one species of *Gymnotus* (*Gymnotus cylindricus*) from Guatemala. The first is protected from predation by its strong electric discharge and the second is geographically isolated from electric eels and other electroreceptive predators such as pimelodid catfishes. Noting that *Brachyhypopomus bennetti*’s monophasic waveform is nearly identical in duration and shape to that of the electric eel, Stoddard (1999) suggested that the convergence may be a form of Batesian mimicry to deter predation by electroreceptive predators that associate monophasic EODs with electric eels.

Our fieldwork confirms that electric eels are common at the *Odontohypopomus* collection localities, as are piscivorous *Gymnotus* species that likely account for some of the tail damage observed in our specimens. Preference for floating meadow habitat near deep water also make these species vulnerable to predation from large pimelodid catfishes, such as *Pseudoplatystoma tigrinum*, a species that may specialize on gymnotiforms (Reid 1983, Zuanon 1990). Exposure to the high predation pressure that likely characterizes Amazonian floating meadow habitat might favor the evolution of an EOD mimic of *Electrophorus electricus* and Stoddard’s hypothesis makes predictions with respect to the behavior of electroreceptive predators that should be tested. However, in our collections we note similar proportions (>60%) of both *Brachyhypopomus bennetti* and *Brachyhypopomus walteri* that exhibit regenerated caudal filaments and posterior anal fin rays from earlier predation. We do not know the identity of these “tail grazers” and what proportion of them are electroreceptive, but the monophasic EOD of *Brachyhypopomus bennetti* clearly does not prevent a high proportion of individuals from suffering such injuries.

**Figure 10.**
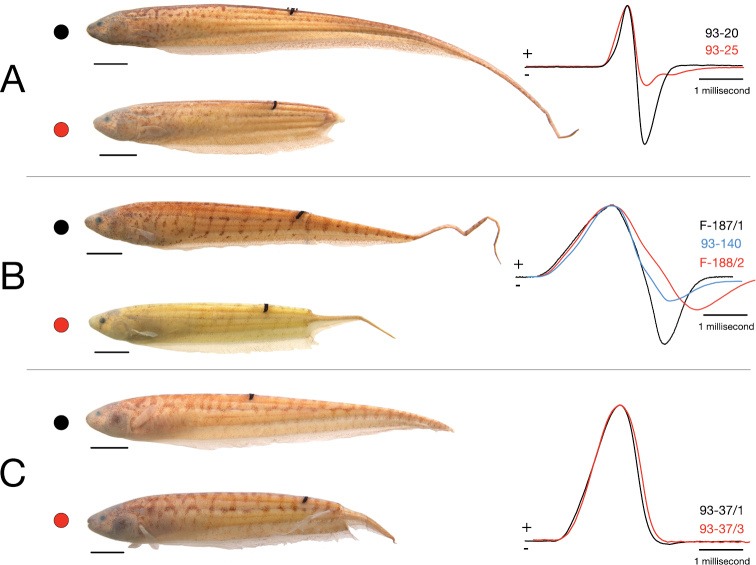
Effect of loss of caudal portion of body by predation on electric organ discharge (EOD) waveform in three species of *Brachyhypopomus*. Undamaged individuals indicated by black dots and black EOD trace, those with regenerating caudal body following substantial injury with red **A** *Brachyhypopomus pinnicaudatus* specimens 93-20 (above) and 93-25 (below) **B** *Brachyhypopomus walteri* paratypes 93-188/2 (above) and 93-187-1 (below). Damaged paratype 93-140 (blue trace) not shown **C** *Brachyhypopomus bennetti* paratypes 93-37/1 (above) and 93-37/3 (below). EODs shown with head positivity upwards and amplitude-normalized.

An alternative (or additional) advantage of EOD monophasy in *Brachyhypopomus bennetti* may be related to the fact that, in contrast to its biphasic relatives, its EOD waveform remains largely unaltered after tail predation (Fig. 10). In a typical biphasic *Brachyhypopomus* EOD, the anterior and caudal portions of the electric organ do not contribute equally to the head-positive and head-negative phases. Only the posterior portion of the electric organ that includes the caudal filament produces the head-negative second phase to the EOD waveform, while the anterior electric organ produces a mostly head-positive, monophasic pulse (Bennett 1961, 1971, Caputi 1999, Stoddard 1999, Stoddard and Markham 2008). For this reason, individuals suffering predation injuries to the caudal filament and caudal portion of the body produce EODs with attenuated head-negative second phases (Fig. 10A, B). Electrical crypsis by biphasy may be effectively impossible under conditions of heavy “tail grazing,” in which case selection may favor other adaptive solutions. EOD monophasy for electric eel mimicry is one interesting possibility, but monophasy for stability of the waveform is another.

Active electroreception and electric communication rely upon “tuberous”-type dermal electroreceptors some populations of which are narrowly tuned to the peak frequency of the fishes’ own EOD (Bastian 1976, 1977, Hopkins 1976) and to the EODs of conspecific individuals detected at a distance (Hopkins and Heiligenberg 1978). Any mismatch between a fishes’ EOD frequency spectrum and the frequency sensitivity of its own electroreceptors (and those of conspecific individuals) will be deleterious. In species with biphasic EODs, tail predation not only decreases the amplitude of the EOD, but alters its waveform and thus its frequency spectrum (Fig. 10A, B), whereas such injury in *Brachyhypopomus bennetti* only affects amplitude (Fig. 10C). Hence it is worth considering that the monophasic EOD of *Brachyhypopomus bennetti* may have evolved to provide its electrosensory system greater robustness to tail injuries. Likewise, the positioning of more of the electric organ rostrally on the body (as opposed to along an exposed caudal filament) and a high amplitude EOD would increase resiliency of electrosensory function in fish that regularly lose caudal electrocytes to predation. These two hypotheses to account for the distinctive characteristics of *Brachyhypopomus bennetti*’s EOD and electric organ are not incompatible: this speciescould, in theory, enjoy advantages from both electric eel mimicry and EOD waveform stability simultaneously.

Sister species *Brachyhypopomus walteri* lacks these characteristics of the electric organ and EOD despite also persisting in floating meadows. However, differences in behavior or feeding ecology of these two species might result in exposure to different selective pressures, even in the same habitat, and future study may yet indicate differences in niche breadth and distribution between *Brachyhypopomus walteri* and *Brachyhypopomus bennetti*. The very different EOD waveforms of these two species may mediate reproductive isolation between them and reproductive character displacement may have also played a role in the divergence of these signals, as has been suggested for sympatric *Gymnotus* species (Crampton et al. 2011).

## Comparative material examined

Type material: ***Brachyhypopomusbeebei*** (Schultz), holotype USNM 120753 and two paratypes MBUCV 15163 and AMNH 15453, Caripito, Venezuela; ***Rhamphichthys artedi*** Kaup, syntype, MNHN 3157, La Mana River, French Guyana; ***Rhamphichthysmulleri*** Kaup, syntype, MNHN 3983 Cayenne, French Guiana; ***Rhamphichthysbrevirostris*** Steindachner, two paralectotypes NMW 650398, Rio Guaporé, Brazil; ***Parupygus savannensis*** Hoedeman, holotype, ZMA 102375, Sipaliwini River, Surinam; ***Parupygus litaniensis*** Hoedeman, holotype, ZMA 100428, Litany River, Surinam, and paratype, ZMA 100407, La Mana River, French Guiana; ***Hypopomus occidentalis*** Regan, 7 syntypes, BMNH 1914.5.18.94–8, Río Condoto, Colombia; ***Hypopomus pinnicaudatus*** Hopkins, holotype, ANSP 163463, and paratype CU 71933, coastal French Guiana; ***Hypopomus janeiroensis*** Costa & Campos da Paz, holotype and paratypes, MZUSP 43130, tributary of the Rio São Joao near Rio de Janeiro, Brazil; ***Brachyhypopomus bullocki***: holotype ANSP 187477, paratypes (15) ANSP 138024 (Colombia: Meta-Orinoco); Type material for ***Brachyhypopomusdiazi*** is unlocatable. New collections of this species were made at the type locality (CU 94862). Non–type material: ***Hypopomus artedi***: CU 71952 & CU 71953 (French Guiana: La Mana); FMNH 56768 (Brazil: Para: Amazon); USNM 225655 (Suriname: Sipaliwini). ***Brachyhypopomus beebei***: ANSP 130488 (Ecuador: Napo); CAS 67702 (Peru: Amazon); CU 71944, CU 71945, CU 71956 (French Guiana: Sinnamary); FMNH 102272 (Peru: Yasuni–Amazon); FMNH 102281 (Peru: Napo–Amazon); FMNH 102279, 102284 & FMNH 102285 (Peru: Aguarico–Amazon); MCZ 30175 (Guyana: Nicaparoo–Amazon); USNM 301694 (French Guiana: coastal drainage); USNM 302003 (Suriname: Nickerie–Corantijn). ***Brachyhypopomus brevirostris***: AMNH 40086 (Bolivia: Beni–Madeira–Amazon); CU 71942 (French Guiana: Kaw); CU 71957 (Venezuela: Apure–Orinoco); FMNH 54544 (Brazil: Guaporé); INPA 4385 (Brazil: Mato Grosso: Aripuaná); INPA 7358–7363, 7366–7367, 7373, 7377, 7379–7382, 7388, 7392 (Brazil: Roraima: Branco–Amazon); UMMZ 204512, 204743 (Bolivia: Beni–Madeira–Amazon); USNM 198081, 306868 (Brazil, Amazonas: Amazon). ***Brachyhypopomus bullocki***: see list in Sullivan and Hopkins (2009); ***Brachyhypopomus diazi***: MBUCV 16895 (Venezuela: Tocuyo); CU 94862 (Venezuela: Yaracuy); CU 94862 (Venezuela: Portuguesa–Orinoco). ***Brachyhypopomus occidentalis***: ANSP 163176 (Costa Rica: Limon); CU 71936 (Panama: Atlantic slope); CU 71934 (Colombia: Pacific slope); FMNH 56791 (Colombia: San Juan); FMNH 56779, 56788, 56789 (Colombia: Pacific slope); FMNH 79124, 93123 (Ecuador: Palenque); INHS 60283 & USNM 121586 (Venezuela: Lago Maracaibo); USNM 302020 (Panama: Río Santa Maria). ***Brachyhypopomus pinnicaudatus***: ANSP 163465 (French Guyana: coastal); FMNH 54546 (Brazil, Rio Grande do Sul: coastal); FMNH 54550 (Brazil, Amazonas: Amazon); INPA 4389, MCZ 78165 (Brazil, Amazonas: Amazon); UMMZ 207598 (Paraguay: Pilcomayo); USNM 216870 (Colombia: Amazon); USNM 229915 (Brazil, Amazonas: Amazon); USNM 263859 (Peru: Madre de Dios–Amazon); USNM 301966, 301967, 306866, 306694, 306749, 306789 (Brazil, Amazonas: Amazon). ***Brachyhypopomus* sp. (indet. or undescribed)**: AMNH 39774, 39932 (Bolivia: Itenez–Amazon), AMNH 78060, 78112, 78114 (Peru: Ucayali–Amazon), ANSP 53894 (Brazil, Mato Grosso: Rio Paraguay); CU 71941 (Guyana: Rupununi); FMNH 53325 (Guyana: Nickaparoo); MCZ 2769 (Brazil, Amazonas: Amazon); MCZ 9435 (Brazil, Para: Amazon); MCZ 30175 (Guyana: Nickaparoo); MCZ 52124 (Brazil, Minas Gerais: São Francisco); UMMZ 206285, 206470 (Paraguay: coastal); USNM 199213 (Brazil, Mato Grosso: Juruena–Amazon); USNM 266718 (Venezuela: Upper Orinoco); USNM 260254 (Venezuela: Apure–Orinoco); USNM 301979 (Brazil, Mato Grosso: Guaporé); USNM 301981 (Brazil, Mato Grosso: Xingu). Additional specimens examined in Sullivan (1997).

## Supplementary Material

XML Treatment for Odontohypopomus

XML Treatment for Brachyhypopomus (Odontohypopomus) walteri

XML Treatment for Brachyhypopomus (Odontohypopomus) bennetti

## Appendix I

Radiographs of *Brachyhypopomus bennetti* sp. n. holotype (top) and *Brachyhypopomus walteri* sp. n. holotype (bottom) (doi: 10.3897/zookeys.327.5427.app1) File format: JPEG image file (jpg).

## Appendix II

Audio recording of resting electric organ discharge of *Brachyhypopomus walteri* sp. n. (doi: 10.3897/zookeys.327.5427.app2) File format: Waveform Audio File (wav).

**Explanation note:** Audio recording of resting electric organ discharge of *Brachyhypopomus walteri* sp. n. holotype specimen (93-219) [.wav file, 4.3 MB].

## Appendix III

Audio recording of resting electric organ discharge of *Brachyhypopomus bennetti* sp. n. (doi: 10.3897/zookeys.327.5427.app3) File format: Waveform Audio File (wav).

**Explanation note:** Audio recording of resting electric organ discharge of *Brachyhypopomus bennetti* sp. n. holotype specimen (93-220) [.wav file, 4.2 MB].
