# Supplementary figures and images for: Two new species and a new subgenus of toothed Brachyhypopomus electric knifefishes (Gymnotiformes, Hypopomidae) from the central Amazon and considerations pertaining to the evolution of a monophasic electric organ discharge
Source: Zookeys. 2013 Aug 28;(327):1–34. doi: 10.3897/zookeys.327.5427 (PMC3807744; doi:10.3897/zookeys.327.5427)

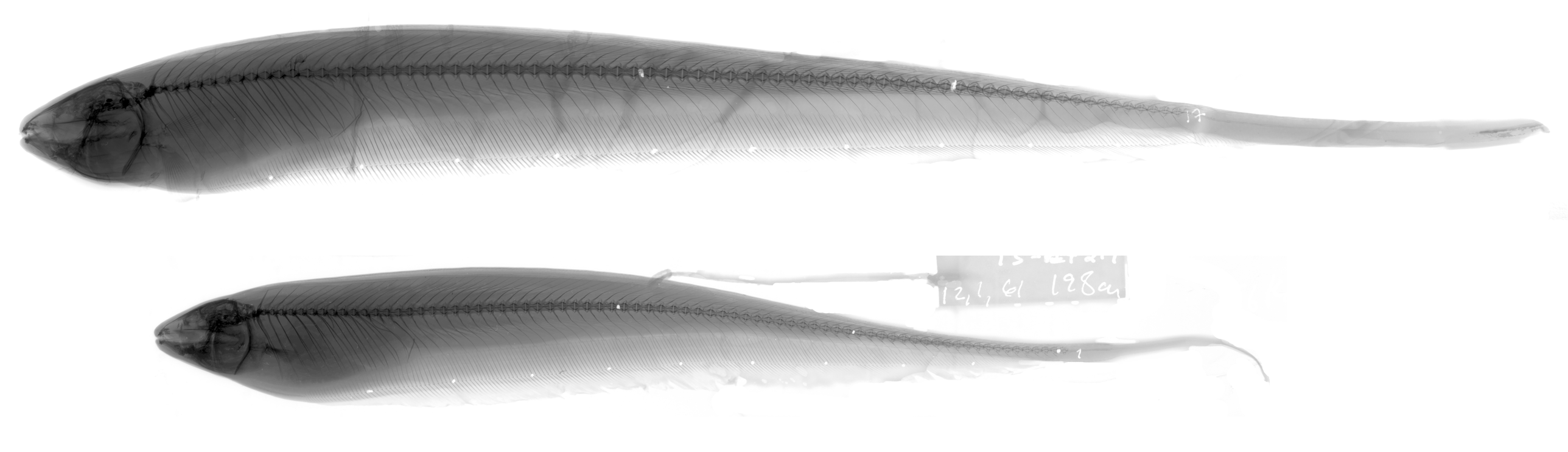

Supplement: Supplementary file 4 — Radiographs of Brachyhypopomus bennetti sp. n. holotype (top) and Brachyhypopomus walteri sp. n. holotype (bottom) (doi: 10.3897/zookeys.327.5427.app1) File format: JPEG image file (jpg). [file ZooKeys-327-001-s001.jpg]
